# Critical behavior in a stochastic model of vector mediated epidemics

**DOI:** 10.1038/srep27202

**Published:** 2016-06-06

**Authors:** E. Alfinito, M. Beccaria, G. Macorini

**Affiliations:** 1 Dipartimento di Ingegneria dell’Innovazione, Università del Salento, Lecce, Italy; 2 Dipartimento di Matematica e Fisica “Ennio de Giorgi”, Università del Salento, Lecce, Italy; 3 INFN (Istituto Nazionale di Fisica Nucleare) Sezione di Lecce - Via Arnesano 73100 Lecce, Italy.

## Abstract

The extreme vulnerability of humans to new and old pathogens is constantly highlighted by unbound outbreaks of epidemics. This vulnerability is both direct, producing illness in humans (dengue, malaria), and also indirect, affecting its supplies (bird and swine flu, Pierce disease, and olive quick decline syndrome). In most cases, the pathogens responsible for an illness spread through vectors. In general, disease evolution may be an uncontrollable propagation or a transient outbreak with limited diffusion. This depends on the physiological parameters of hosts and vectors (susceptibility to the illness, virulence, chronicity of the disease, lifetime of the vectors, *etc.*). In this perspective and with these motivations, we analyzed a stochastic lattice model able to capture the critical behavior of such epidemics over a limited time horizon and with a finite amount of resources. The model exhibits a critical line of transition that separates spreading and non-spreading phases. The critical line is studied with new analytical methods and direct simulations. Critical exponents are found to be the same as those of dynamical percolation.

The study of epidemic outbreaks is a largely interdisciplinary topic. It exploits cross-fertilization among different scientific areas, like physics of complex systems, epidemiology, and microbiology. The ability to understand when a definite disease diffuses or extinguishes is crucial for setting up an efficient strategy of prophylaxis and vaccination with the aim of preventing or stopping the infection diffusion^1^. Specific information about the pathogen is mandatory. In particular, we need to know the way it affects the hosts and how it diffuses. One of the most interesting transmission strategies developed by pathogens is the vector-mediated epidemics (VME)^1^. This mechanism can be found acting among static individuals like trees or vines (olive quick decline syndrome (OQDS), Pierce Disease) and among mobile individuals (Nile fever, yellow fever, malaria, dengue, chikungunya, bluetongue disease, *etc.*). In the case of mobile populations, it is unclear what the evolutionary mechanism is behind the VME strategy^2^,^3^. Vector-mediated epidemics are generally very aggressive and difficult to eradicate. Their diffusion in Europe – mainly due to global warming – is an emerging concern^4^. On the side of prevention and curing of VME, the adopted strategies are quite complex and more diverse than those in direct transmission epidemics. In general, dual action is necessary by reducing the number of infected vectors (prophylaxis) and the subset of susceptible individuals (vaccination). These two actions may be expensive and not completely efficient on their own. The optimal mixed strategy is not known *a priori* and definitely depends on the specific characteristics of the pathogen and vector, like virulence and mortality. Therefore, it is highly desirable to devise simplified models that captures something of the real world. Here, we focus on such a model to describe the critical behavior on a limited time horizon with finite size resources. Also by using these constraints, the model reveals some interesting features. In particular, a finite size scaling analysis will show that the critical behavior is the same as in dynamical percolation models. We stress that moving from our ideal mathematical modeling to the real world, more information but also more caution has to be made in the *one-to-one* interpretation and use of the results. A very relevant issue concerns the ethical strategies to adopt about vaccination and prophylaxis in humans with respect to animals or plants. Our analysis just suggests qualitatively what could be right directions from the point of view of efficiency, while actual intervention policies must take into accounts other fundamental and more realistic aspects of the problem.

## Lattice Models for Epidemics and Critical Behavior

About one century ago, after the first discrete-time models by Hamer^5^ and Ross^6^, the SIR model for epidemics was introduced^7^. In this model, the individuals in the population may be in three states (compartments): susceptible (S), infected (I), and recovered (R). The number of individuals in each compartment is a function of time, and the system is governed by a coupled set of ordinary differential equations (ODE). The SIR model exhibits a phase transition between non-spreading (NS) and spreading (S) regions^7^. Later, a variety of other models^8^,^9^,^10^,^11^,^12^,^13^ were developed to cope with different specific epidemic conditions. Notable are the so-called contact models, particularly the Susceptible-Infected-Susceptible (SIS) model, whose critical properties have been widely analysed^8^,^9^,^10^,^11^,^14^,^15^. Although the ODE-based approach can predict some relevant results, it does not capture the spatial structures of outbreaks. Stochastic lattice models are discrete *automata* that overcome this limitation. The individuals are associated with sites of a regular or complex network. Simple elementary transition rules lead to non-trivial collective behavior. The original ODE models are recovered as a mean field approximation. Although lattice models significantly improve over ODE models, they are somewhat artificial and involve features that are not directly related to the real world, like lattice topology. One could be concerned that they are too simplified to be an accurate description. This is strictly true in general, but not when one focuses on the critical behavior – *i.e.* its dependence on the finite size. What saves the day is Kadanoff idea of *universality*^16^. Correlations in space and time become more and more important as we approach a critical phase transition. The effective degrees of freedom of the lattice system partially delocalize, and microscopic details become more and more irrelevant. Thus, the critical behavior is characterized by a small set of quantities, like critical exponents and amplitude ratios, that may be computed in quite simple models identifying the so-called *universality classes*^17^. The SIR/SIS epidemic models belong to different classes: those of dynamical^14^,^18^,^19^ and directed percolation^14^, respectively.

### VME by coupled SIR-SIS models

**Figure 1:**
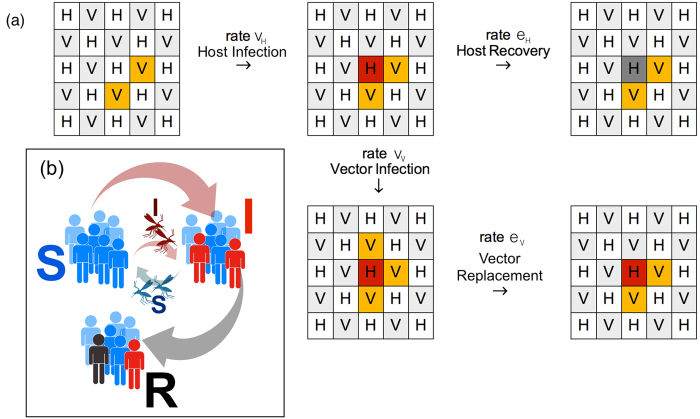
Pictorial description of the SIR-SIS interaction in the VME model. (**a**) Evolution rules illustrated for a particular lattice configuration. Susceptible hosts and vectors are presented in light gray and white, infected sites are red (host) and yellow (vectors), and recovered hosts have a dark gray background. The rates *v*_*V*_ and *e*_*H*_ govern host infection (through the contact interaction with infected vectors) and recovery. The rates *v*_*H*_ and *e*_*V*_ determine the host-mediated vector infection, death, and replacement. (**b**) Pictorial representation of the interaction between the SIR and SIS models for host (represented as humans) and vectors (mosquitos).

In order to describe the spreading of VME, we adopt the recently introduced coupled SIR-SIS model^10^, which we simply refer to as the VME model. The SIR and SIS dynamics interact in the spreading mechanism, like in real life. This model captures most of the relevant mechanisms of epidemic spreading, although in a schematic way. The VME model involves two different players, hosts (H) and vectors (V). Only a cross mechanism of infection is available (catalytic infection). Hosts follow SIR evolution, while vectors are governed by SIS dynamics. This is inspired by the idea of two species with very different life expectancies. Hosts and vectors are placed on a regular checkerboard (see^20^ for epidemics in complex networks). Self-interactions are absent, and the evolution of one subspecies is always mediated by another species in neighborhood. Susceptible players of each species are infected with a specific *virulence* rate. The model is schematically illustrated in Fig. 1.

As we briefly mentioned in the introduction, most studies point out the global-warming-induced increase in vector populations and in pathogen virulence as one of the main reasons for recent epidemic outbreaks and interest (see, for instance^21^,^22^,^23^,^24^). The SIS component present in our formulation implies instead a one-to-one reproduction of vector units. Thus, our modeling would apply to a local, closed population within a limited time horizon and with limited vital resources available (such as water, or any other reproduction/incubation media).

The different rates of infection for hosts and vectors are denoted by *v*_*H*_ and *v*_*V*_ respectively, which generally differ. The effect of host vaccination is implemented by reducing the rate *v*_*H*_. Vectors are assumed to die more rapidly than hosts. After being infected, they do not recover but die and are reintroduced in the game as susceptible players, with *exit* rate *e*_*V*_. The effect of prophylaxis is to increase this rate and reduce the number of infected vectors. Finally, the infected hosts may be put out of the game (recover or die) with an assigned exit rate, *e*_*H*_. For a small exit rate, hosts tend to remain in the infected state, and their illness becomes chronic. In contrast, a large exit rate reduces the number of individuals that they can infect. Note that the rate of exit may also be associated with a prophylactic practice, like the eradication of infected trees in VMEs like OQDS^25^. Tuning these ratios up or down leads to winning of the spreading or extinguishing phases. A list of the aforementioned rates and of some other useful symbols is reported in Table 1.

### Infection paradigms

To reduce the number of parameters, we adopt the condition *v*_*H*_ − *e*_*H*_ = *v*_*V*_ − *e*_*V*_, which represents the equality of the effective rates of spreading for each species^10^. There is no deep epidemiological reason behind this condition that is a mere simplifying assumption. In principle, our analysis could be extended to the more involved 3-parameter case. Anticipating our findings, we shall see that each point of the critical line will be equivalent from the point of view of critical behavior being characterized by the same exponents governing its size dependence – although with varying critical amplitudes. Switching on more parameters, as it would be in a realistic model, replaces the critical line by a critical surface where the same features are expected. In our setup, the independent variables reduce to the ratios *e*_*H*_/*v*_*V*_, *v*_*H*_/*e*_*V*_, and the epidemic spreading can be explored in the plane of *p* = 1/(1 + *e*_*H*_/*v*_*V*_) and *h* = 1/(1 + *v*_*H*_/*e*_*V*_). For each specific ratio of the host exit and vector virulence rate, it is possible to find a corresponding ratio of the vaccination and prophylaxis rates sufficient to counteract the spreading. Conversely, the non-spreading region NS is reached for each value of *h*, making the ratio *e*_*H*_/*v*_*V*_ larger than its critical value. In a realistic context, this view would be more significant in VMEs with non-human hosts, such as OQDS or Pierce Disease, for which vaccination is still not allowed and improving the host exit rate is an admissible prophylactic strategy. In other words, in order to confine the epidemics, it is enough to make the exit rate (eradication) just a little bit larger than (1/*p*_*c*_ − 1)*v*_*V*_.

## Results

### The critical line

**Figure 2:**
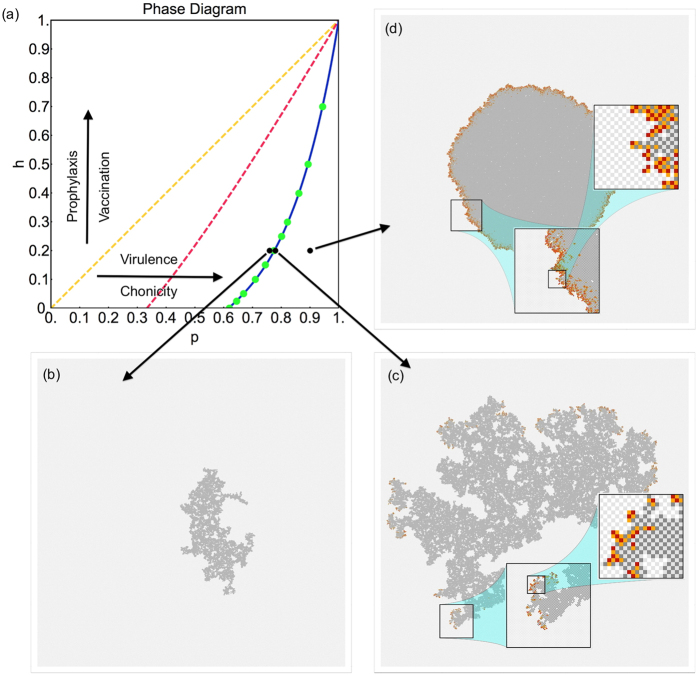
Phase diagram and epidemic spreading. Panel (**a**): Phase diagram of the VME model with the critical lines in various approximation schemes: MF (dashed yellow), PMF (dashed red), IPMF (blue). Green dots are exact critical points extracted from our Finite Size Scaling analysis of Monte Carlo data, in agreement with^10^. Panels (**b**–**d**) display typical realizations of the epidemics on a 1000^2^ lattice for three different values of *p* = 0.76, 0.78, 0.9, and *h* = 0.2. Susceptible hosts and vectors are light grey and white dots, infected hosts and vectors are red and orange. In more detail, panel (**b**) describes a point near the critical line, but in the non-spreading phase. The cluster of recovered sites is shown after the epidemics terminates. Panel (**c**) shows what happens at the transition line. The snapshot is taken at an arbitrary intermediate time. At later times, the epidemics wrap around the periodic boundary conditions and eventually stop, due to the available finite number of sites. Finally, panel (**d**) shows the typical evolution deep in the spreading phase, again shown at intermediate time. The magnification boxes show the details of the checkerboard structure of the model in the active regions of the epidemic. More details about the specific features of these realizations are discussed in the main text.

The continuous set of critical points (*p*_c_, *h*_c_) constitutes the critical line of the model (see Fig. 2). The analysis of^10^ is based on the Master Equation formulation of the stochastic dynamics:


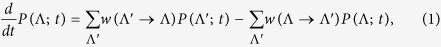


where *P*(Λ; *t*) is the probability that the full-lattice VME model is in the state Λ at time *t*, and *w*(Λ → Λ′) are the rates of the configuration change Λ → Λ′. Assuming invariance under translations, the Master Equation can be turned into an infinite coupled system of evolution equations for the joint probabilities *P*(*s*_1_, *s*_2_, …) for finding a cluster of neighbouring sites in the states *s*_*i*_. The stochastic model is thus substantially beyond any simple description that does not take into account fluctuations on the microscopic scale. In^10^, an approximate critical line has been determined by a stability analysis of the Master Equation. This required a closure hypothesis to deal with a finite number of evolution equations. The mean-field (MF) approximation corresponds to the uncorrelated choice *P*(*s*_*i*_, *s*_*j*_) = *P*(*s*_*i*_) *P*(*s*_*j*_). The pair mean field (PMF) line is obtained by replacing the three-site probabilities with the combination 
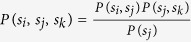
. In the MF case, the evolution of the system is truncated to a set of three coupled differential equations, while in the PMF case gives a system of five equations. For the analytical determination of the critical line, see Methods A. In panel (a) of Fig. 2 it is represented by the yellow (MF) and red (PMF) curves. Neither approximation is accurate in reproducing the data points (green circles) obtained by Monte Carlo simulation^10^. We have further improved this approximation scheme by introducing two arbitrary numerical coefficients 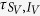, 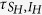 (IPMF) appearing in the closure relation.


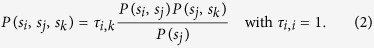


The factors *τ*_*i*,*k*_ are regarded as phenomenological parameters that encode the effect of the higher joint probabilities. In principle, they may be determined by comparison with Monte Carlo simulation data^26^. The best improved critical line is obtained by the choice 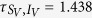, 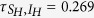 and is shown by the blue curve in Fig. 2. Remarkably, all data points are accurately fit by our simple two-parameter modification.

**Figure 3:**
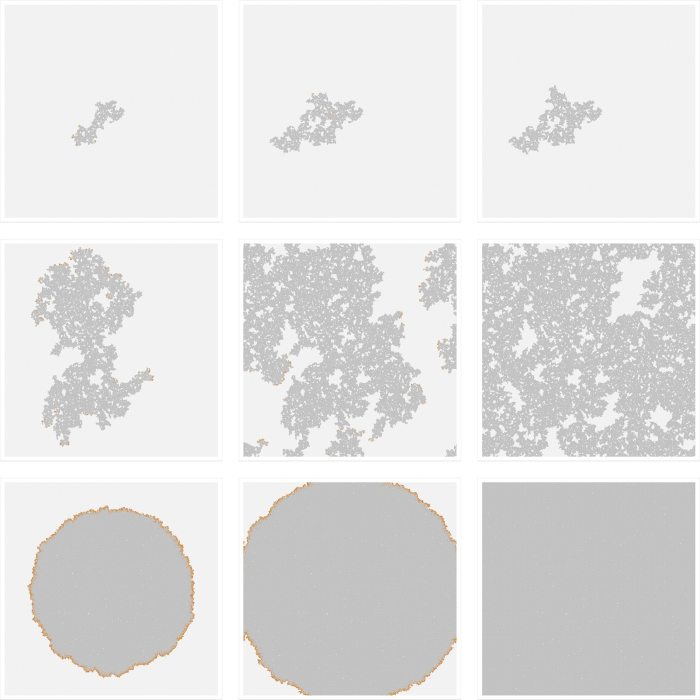
Temporal evolution at specific points in the (*p*, *h*) plane. We show three snapshots taken during the temporal evolution of the VME model on a 1000 × 1000 lattice at the three points *p* = 0.76, 0.78, 0.9 (rows from top to bottom), and *h* = 0.2, as considered in Fig. 2. The first row is in the non-spreading phase. After the initial outbreak, the number of infected hosts initially grows and then reduces, leading to a final static state where the cluster of recovered hosts has not reached the lattice boundary. The second row is on the critical line. The infection invades the lattice, wraps around the periodic boundary, and eventually terminates. The final cluster of recovered hosts is fractal due to isles of different shapes containing untouched susceptible hosts surrounded by recovered ones. Finally, the third row shows the evolution deep in the spreading phase. Now, the evolution leaves behind a compact cluster of recovered hosts. In the final state, the lattice is almost filled with such sites.

Panels (b–d) of Fig. 2 give a qualitative idea of the general spatio-temporal characteristics of the VME epidemics in different regions of the (*p*, *h*) plane. This is illustrated by snapshots of typical realizations of the epidemics on a 1000 × 1000 lattice. Susceptible hosts and vectors are represented by light gray and white pixels, while infected hosts and vectors are represented by red and orange pixels, respectively. In panel (b), we consider a point near the critical line but in the non-spreading phase. We show the cluster of recovered sites (RSC) at the time where the epidemics stop. The cluster has a fractal structure and does not reach the lattice boundary. In panel (c), we are precisely on the transition line. The RSC is yet again fractal and shown at an arbitrary intermediate time. At later times, the periodic conditions push the cluster to wrap around the boundaries until the epidemic stops. Finally, in panel (d), we show a realization associated with a point well within the spreading phase. Again, we show the RSC at an intermediate time. The cluster is now compact with an approximately circular shape. The geometrical structure of the infected sites changes as we move off the critical line. At criticality in panel (c), there are small disconnected clusters of infected hosts and vectors at the boundary of the growing cluster of recovered hosts. Instead, deep in the spreading phase in panel (d), these clusters merge in a connected ring along the boundary. A quantitative separation of the spreading and non-spreading phases requires a detailed finite size scaling analysis, to be given later, see equation (3). For more details about the temporal evolution, see Fig. 3. Additional information related to the reproductive ratio *R*_0_ = *P*(*S*_*H*_|*I*_*V*_) *P*(*S*_*V*_|*I*_*H*_) is discussed in Methods A.

### Finite Size Scaling data analysis and critical exponents

The evolution of the VME model is investigated by using a Monte Carlo simulation on a checkerboard square lattice with *L* × *L* sites, assuming periodic boundary conditions. We start with one infected host at the centre of the lattice and let the infection evolve until there are no more infected sites. This is an inactive (adsorbing) state. For each realization of this stochastic process, we measure various quantities of interest. The order parameter *P* is defined to be one if the infection reaches the lattice boundary and zero otherwise. The number *N*_*H*,*R*_ is the total number of recovered hosts when the infection stops (which always happens in finite volume). These static quantities are averaged over all realizations, leading to 〈*P*〉 and 〈*N*_H,R_〉 which are functions of *L*, *p*, *h*. Following^18^, we also introduce the important combination 
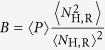
. This is analogous to Binder’s cumulants^27^ and may be shown to be scale invariant. During the simulation, we also record the number of infected hosts *N*_H,I_ when a configuration reaches the boundary, along with the elapsed time *T* at that moment. The averages 〈*N*_H,I_〉 and 〈*T*〉 are computed by restricting to realizations with a spreading infection. We explore the critical line by fixing *h* and varying *p*. For each value of *h*, we expect to find a critical *p*_c_ (*h*) where the spreading-non-spreading transition occurs. From Finite Size Scaling FSS theory (e.g.,^28^), we expect the following laws:


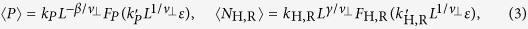


where *ε* = *p* − *p*_c_ (*h*), *β*, *γ*, and *ν*_⊥_ are universal critical exponents, and the coefficients *k*_*P*_, 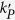, *k*_H,R_, and 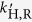 are non-universal metric factors that are expected to be dependent on the spectator coupling *h*^29^. *F*_*P*_ and *F*_H,R_ are universal finite-size scaling functions in the sense that their dependence on *L*, *p*, *h* is fully encoded in the indicated variables. A similar relation holds for the cumulant *B*, but since *B* is scale invariant, it has no *L*-dependent pre-factor and its FSS relation is:


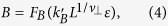


where the value of *B* at *ε* = 0 is also universal. The meaning of equations (3) and (4) is rather simple. For an infinite system, the space correlation length *ξ* diverges like 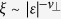. In finite size *L*, the free energy depends on the coupling through the ratio *L*/*ξ* up to a global power of *L* providing the right dimension of the observable quantity under consideration. In general, equations (3) and (4) are empirically valid in a wide region around the transition point *ε* = 0, but scaling corrections are typically present, although subdominant for large *L*. To determine *p*_c_ (*h*) and the exponents, we exploit equation (3) because, for *ε* = 0, it predicts a simple linear behavior between the logarithm of the scaling quantity and log *L*. An alternative method to locate *p*_c_ (*h*) is based on equation (4). It implies that the curves plotting *B* as a function of *p* at different values of *L* intersect precisely at the point *ε* = 0.

As mentioned, we analyze the critical line by evaluating the (constant) exponents in equations (3) and (4) at different values of *h* with a suitable choice of the (technically *non-universal*, *i.e.* model dependent) metric constants *k* and *k*′.

**Figure 4:**
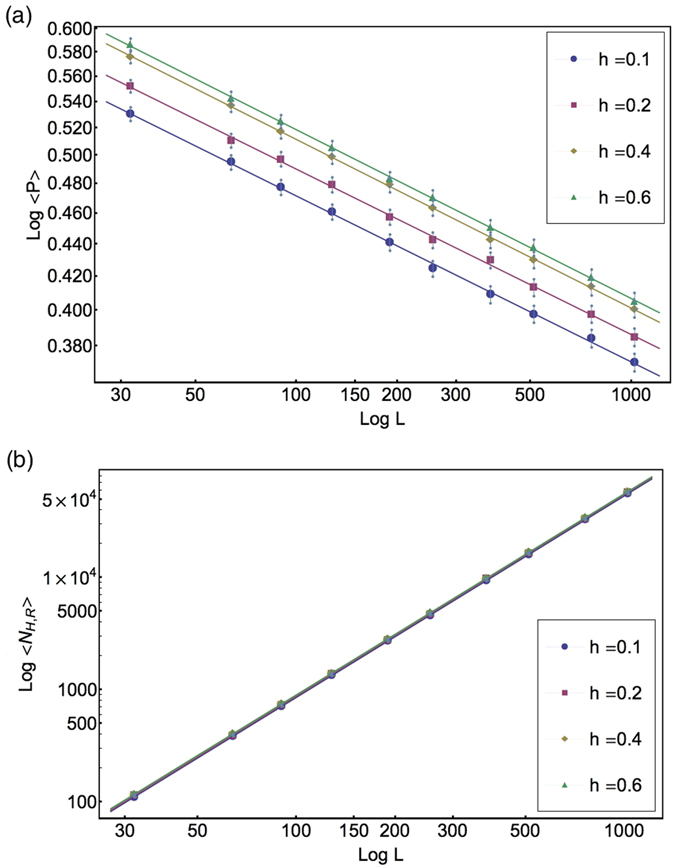
Determination of the critical exponents from scaling at criticality. The order parameter 〈*P*〉 (**a**) and the number of recovered hosts 〈*N*_*H*,*R*_〉 (**b**) at the critical point *p*_c_ (*h*) for the four considered values of *h*. According to the scaling forms in equations (3) and (4), the critical exponents *β*/*ν*_⊥_ and *γ*/*ν*_⊥_ may be extracted from the slope of the data in log-log scale and are clearly independent on the value of the coupling *h*. The error bars in the second plot are smaller than the dots.

**Figure 5:**
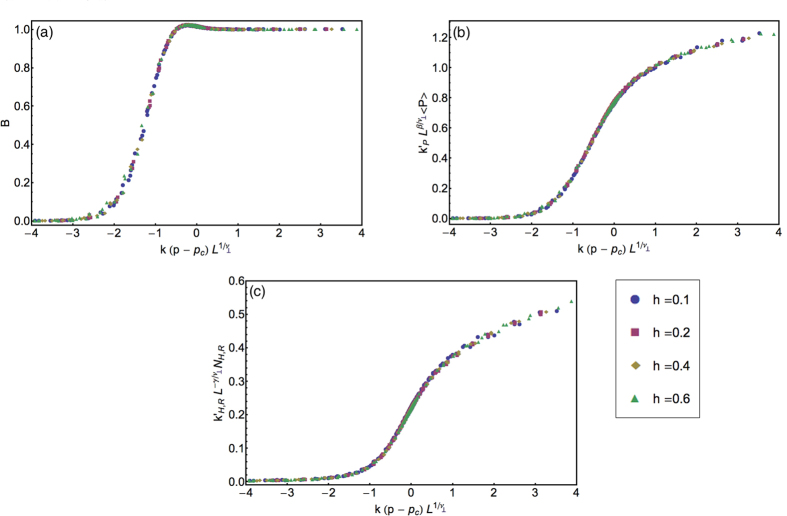
Data collapse of the scaling functions. Data collapse for the quantities *B*, 〈*P*〉, and 〈*N*_*H*,*R*_〉, in panels (**a**–**c**), respectively. With a suitable choice of the *non-universal* metric factors appearing in equations (3) and (4), all data collapse on three smooth curves independent on the coupling *h*. This confirms the *h*-independence of the three scaling functions *F*_*P*_, *F*_H,R_ and *F*_*B*_.

We have run the VME model on lattices with sizes up to 1024 and a typical number of 10^7^ realizations. The analysis has been repeated at the four values *h* = 0.1, 0, 2, 0.4, 0.6, *i.e.*, a growing rate of vaccination for an assigned rate of prophylaxis, or equivalently, a growing rate of prophylaxis for an assigned rate of vaccination. In Fig. 4, we show the linear behavior of the quantities log〈*P*〉 and log〈*N*_H,R_〉 plotted as functions of log *L* for the four choices of *h*. Our best fit results for the critical points and exponents are reported in Table 2. The universal exponents are the same along the critical line. Their values enable us to conclude that, concerning the exponents, the present VME model belongs to the same class of the SIR model^18^. Precisely, we find values of *β*/*ν*_⊥_ and *γ*/*ν*_⊥_ that are in good agreement with the known values 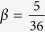, 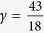, 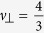 for Dynamical Percolation (DynP)^15^. The same agreement is also found for the cumulant *B*, which was precisely measured to be *B* = 1.0167(1)^18^. A second test is shown in Fig. 5, where we show the collapses of the data for the four values of *h*, according to equations (3) and (4). For any value of *h* the data collapse in a smooth curve, confirming the scaling hypothesis. Moreover, with a suitable choice of the metric factors, the four curves overlay precisely, giving strong support to the universality of the three functions *F*_*P*_, *F*_H,R_, and *F*_*B*_. Comments about the dynamical critical exponents can be found in Methods B.

## Discussion

We have improved the determination of the critical line exhibited by the VME model proposed in^10^ and identified the character of the transition along the whole line, showing that the model has the critical exponents as standard dynamical percolation. In our setup, the two rate ratios *p* and *h* play similar roles, and each point corresponds to a specific disease or specific prophylactic practices. Hence, the asymmetric shape of the critical line may suggest that to improve the epidemic confinement, improving *h* could be more efficient rather than reducing *p*. To give an interpretation, this means that prophylactic practices acting on the vectors (the rate *e*_*V*_) may be more efficient than prophylactic practices acting on hosts, like eradication of trees in the case of the OQDS or Pierce Disease infection. Of course, to make concrete these remarks one should consider more realistic and deep modelizations that are not limited to the analysis of the critical dependence on finite resources, being just one facet of the problem. Then, it should become necessary to account for the actual costs of the different counteractions. Besides, in a real context, ethical problems are a major issue and must be taken into account. From this point of view, any suggestion for a realistic public health policy will definitely separate human infections and agricultural ones, since ethical issues and admissible interventions in the two cases are completely different. As we stressed, this is out of reach for our VME model. Nevertheless, from the point of view of the critical behavior, we have shown the irrelevance of the SIS sub-dynamics. The VME model is actually a SIR model in disguise. Similar conclusions are expected to hold for analogous models appearing in completely different applications, like in the context of internet viral diffusion^30^, opinion dynamics^31^, or traffic simulation^32^.

## Methods

### Determination of the critical line

The evolution of the VME model can be derived from the Master Equation in terms of a system of hierarchical differential equations describing the time evolution of translation invariant *n*-sites joint-probabilities. The equations for *n*-sites probabilities involve the (*n* + 1)-sites ones. If we consider the simplest 1-site probabilities *P*(*i*), defined as the mean value of the Kroneker *δ*-function, *P*(*i*) = 〈*δ*(*σ*_*j*_, *i*)〉, then their evolution equations read


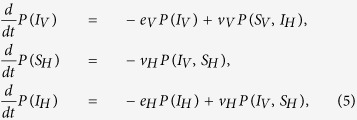


where *S*_*V*_, *I*_*V*_ denote a susceptible and infected vector, while *S*_*H*_, *I*_*H*_, *R*_*H*_ denote susceptible, infected, and recovered hosts. The 2-sites probabilities are similarly defined as the mean value of the product of two neighbouring sites delta function. The equations for *P*(*S*_*V*_) and *P*(*R*_*H*_) follow from the normalization of the probabilities *P*(*S*_*V*_) + *P*(*I*_*V*_) = 1 and *P*(*S*_*H*_) + *P*(*I*_*H*_) + *P*(*R*_*H*_) = 1. The first line in equation (5) simply expresses the fact that the probability *P*(*I*_*V*_) for a vector to be infected decreases according to the exit rate for vectors *e*_*V*_, and increases through contacts with infected hosts. The gain term is proportional to the probability of finding the vector in the susceptible state, with a first neighbouring infected host. The interpretation of the other two lines in equation (5) is similar. If we include the next level in the hierarchy, *i.e.* the equations for the 2-sites probabilities, the system becomes


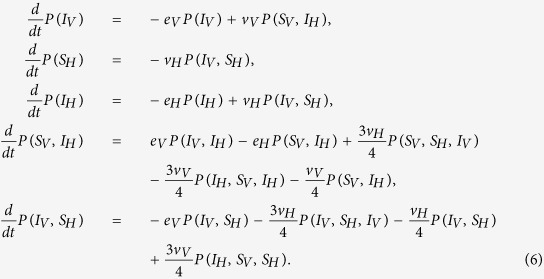


As anticipated in the main text, the two standard closure schemes of the system are the MF or a PMF approximation. Here, we present our improved IPMF scheme based on Equation (2). The determination of the critical line follows from the analysis of the linearized problem around an exact solution with a small number of homogenously distributed infected sites. We introduce the following notation for the five independent 1-site and 2-sites probabilities: *P*(*I*_*V*_) = *x*(*t*), *P*(*S*_*H*_) = *y*(*t*), *P*(*I*_*H*_) = *z*(*t*), *P*(*I*_*V*_, *S*_*H*_) = *v*(*t*), *P*(*S*_*V*_, *I*_*H*_) = *u*(*t*). In the MF case the analysis is straightforward, and the critical line corresponds to the line *p* = *h* in the (*p*, *h*) plane^10^. In the IPMF scheme, starting from Equation (6), the linearized problem reduces to the following set of differential equations


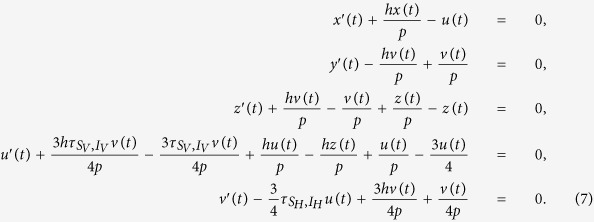


The critical line is then determined from the analysis of coefficient matrix for the linearized problem:


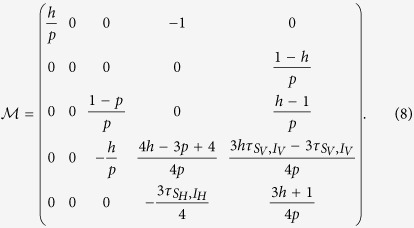


The stability line separating the spreading/non-spreading phases of the epidemic is then obtained setting to zero the largest eigenvalue of the matrix (discarding trivial solutions); this gives a relation between *h* and *p* which can be written as


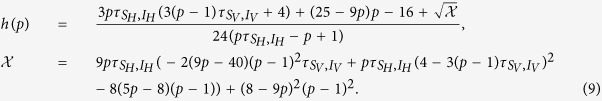


For *τ*_*i*,*j*_ = 1, we recover the results of^10^. From the point of view of the time evolution of the system, the two non-spreading and spreading phases may be characterized by looking at the reproductive ratio


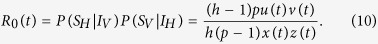


A study of the explicit numerical solutions of the system (6) shows that, as expected, all epidemics end in a finite time in both phases, reaching a stationary states without infected hosts or vectors. The temporal evolution of *R*_0_(*t*) is non trivial. In the non-spreading phase, *R*_0_(*t*) drops monotonically to a constant asymptotic value <1, and its first derivative is negative and monotonic as well. On the other side, in the spreading phase, *R*_0_(*t*) shows an almost constant plateau, with *R*_0_(*t*) > 1, during the initial spreading of the epidemic, and eventually drops as soon as the stationary state is reached. The fact that all epidemics can be eradicated within a finite time horizon is strictly due to the fact that we simulate in finite size, *i.e.* with a local closed population. This would be an unsound assumption for the realistic all-round study of widespread and global endemic infections such as malaria, dengue and various others. In the present context, this is not a problem because we are only focusing on the study of the size dependence and need to extrapolate from finite size data. The study of the fluctuations of the quantities appearing in equation (10) in the lattice stochastic model is an interesting issue that could provide improved estimators for the spreading/non-spreading transition, but is beyond the scope of the present study.

### Dynamical exponents

Apart from the static critical exponents, the DynP class is also characterized by a further dynamical exponent. This is defined at criticality in infinite size *L* → ∞ by the temporal evolution of the growing lattice of recovered hosts. This dynamical exponent appears in the scaling law 〈*T*〉 ~ *L*^*z*^, where *z* = *ν*_‖_/*ν*_⊥_ measures the ratio of correlation exponents in time and space^33^,^34^,^35^. A precise measure of *z* may be found in^36^ and reads *z* = 1.1309(3). Similarly, one expects that 〈*N*_H,I_〉, which is on the boundary of the recovered cluster, scales with *L* with exponent *d*_*F*_ − *z* = 0.7649(1) at the time when the boundary is reached. Here 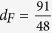 is the fractal dimension of the recovered hosts cluster. Our analysis is focused on the static, *i.e.* spatial, critical properties and is less precise concerning dynamical quantities. From our data for 〈*T*〉, we obtain *z* = 1.13(1) and *d*_*F*_ − *z* = 0.76(9) for the scaling of 〈*N*_H,I_〉, in fair agreement with the expected values^37^.

## Additional Information

**How to cite this article**: Alfinito, E. *et al.* Critical behavior in a stochastic model of vector mediated epidemics. *Sci. Rep.* **6**, 27202; doi: 10.1038/srep27202 (2016).

## Figures and Tables

**Table 1:** Index of main symbols used in the text.

Symbols	Definitions
*v*_*H*_, *v*_*V*_	rates of infection for hosts (H) and vectors (V)
*e*_*H*_, *e*_*V*_	exit (die, recovery, or replacement) rates for hosts (H) and vectors (V)
*p*	virulence/chronicity parameter *p* = 1/(1 + *e*_*H*_/*v*_*V*_)
*h*	prophylaxis/vaccination parameter *h* = 1/(1 + *v*_*H*_/*e*_*V*_)
*L*	linear size of the model
*T*	time needed for the infection to reach the lattice boundary
*P*	order parameter defined as 1 if a realization of the infection reaches the lattice boundary and 0 otherwise
*N*_H,R_	total number of recovered hosts when the infection stops
*B*	Binder cumulant - like combination, useful to identify the critical line
*β*, *γ*, *ν*_⊥_	various critical exponents governing the universal behavior. Their theoretical values in the dynamical percolation class are: 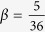 , 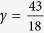 , 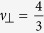

In this table, we list the main symbols used in the text, recalling their definitions.

**Table 2:** Numerical results and critical exponents.

	*h* = 0.1	*h* = 0.2	*h* = 0.4	*h* = 0.6
*p*_*c*_	0.7110(8)	0.7752(8)	0.86213(5)	0.92072(6)
*β*/*ν*_⊥_	0.104(1)	0.104(2)	0.1042(2)	0.1041(9)
*γ*/*ν*_⊥_	1.79(4)	1.79(3)	1.796(8)	1.793(3)
B(*p*_*c*_)	1.016(4)	1.016(6)	1.015(9)	1.0167(1)

In this table, we summarize the results for our best estimates of the critical points, critical exponents *β*/*ν*_⊥_, *γ*/*ν*_⊥_, and the values of *B* at the critical point for the four considered values of *h*. For comparison, the theoretical values are 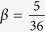, 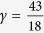, 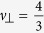 giving respectively 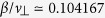 and 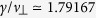. The numerical data from the Monte Carlo simulations are in perfect agreement with the expected values for the Dynamical Percolation class. The value of *B* at the critical point is expected to be *B* = 1.0167(1)^18^ and our results confirm it.
